# Acceptance checklist for clinical effectiveness pilot trials: a systematic approach

**DOI:** 10.1186/1471-2288-13-78

**Published:** 2013-06-13

**Authors:** Georgina Charlesworth, Karen Burnell, Juanita Hoe, Martin Orrell, Ian Russell

**Affiliations:** 1 Research Department of Clinical, Educational and Health Psychology, University College London, 1-19 Torrington Place, London WC1E 7HB, UK; 2 Research and Development Department, North East London NHS Foundation Trust, Goodmayes Hospital, Barley Lane, Ilford, Essex IG3 8XJ, England; 3 School of Health Sciences and Social Work, James Watson (West), 2 King Richard 1st Road, Portsmouth PO1 2FR, England; 4 UCL Mental Health Sciences Unit, University College London, Charles Bell House, 67-73 Riding House Street, London W1W 7EJ, UK; 5 West Wales Organization for Rigorous Trials in Health, Swansea University College of Medicine, Singleton Park, Swansea SA2 8PP, Wales

**Keywords:** Pilot trial, Internal pilot, Randomized controlled trial, Feasibility, CONSORT, ACCEPT, SHIELD, Carer supporter programme

## Abstract

Conducting a pilot trial is important in preparing for, and justifying investment in, the ensuing larger trial. Pilot trials using the same design and methods as the subsequent main trial are ethically and financially advantageous especially when pilot and main trial data can be pooled. For explanatory trials in which internal validity is paramount, there is little room for variation of methods between the pilot and main trial. For pragmatic trials, where generalisability or external validity is key, greater flexibility is written into trial protocols to allow for ‘real life’ variation in procedures. We describe the development of a checklist for use in decision-making on whether pilot data can be carried forward to the main trial dataset without compromising trial integrity. We illustrate the use of the checklist using a pragmatic trial of psychosocial interventions for family carers of people with dementia as a case study.

## Introduction

Pilot studies focus on the science, process, management and resources of planned studies [1]. Their purpose is to increase the likelihood of success of interventions in subsequent larger studies by ensuring they are appropriate and effective in practice [2-4]. Pilot studies help to justify the investment of money and time in those subsequent studies [5]. Hence they are often a requirement of funding bodies [6].

The aims of this article are to: (1) consider the nature and extent of permissible changes to procedures between the pilot and main trial without breaching methodological integrity (2) propose a checklist of areas where amendments to trial procedures may arise (3) illustrate the process of deciding whether to accept data from pilots into main trials.

### Background

Pilot *trials* have been defined as “miniature versions of full trials conducted to ensure the detailed design, methods and procedures are all robust and fit for purpose” [7]. The term ‘pilot trial’ should not be confused with the term ‘pilot study’. The latter has been used synonymously with the term ‘feasibility study’ in which trial processes, resources, management and scientific factors are scrutinised in order to facilitate the planning of large-scale investigations [8].

Recent reviews have highlighted a variety of situations where the term ‘pilot trial’ has been used, or, some would argue, misused. For example: as a substitute for hypothesis-testing studies rather than being preparation for a full trial; to make inadequately powered studies sound more attractive to publishers; and, to report on outcomes rather than design, methods and procedures [2,9,10].

Having established the definition of pilot trials as ‘full trials in miniature’ we next need to define the common subtypes of pilot trial, namely the internal and external pilot. Lancaster and colleagues define ‘external’ pilot trials as ‘stand alone’ pieces of work, planned and conducted independently of the main trial [6]. In contrast ‘internal’ pilot trials are set up with the intention of being incorporated into the main trial. Lancaster *et al*. described the purpose of pilot trials as being limited to collection of data for sample size re-calculation such that pilots do not allow for the pre-testing of the feasibility of other factors relating to the trial. However, we suggest that a degree of feasibility testing should be allowable in the internal pilots of pragmatic trials, and that scientific integrity can be maintained as long as the degree of variation to procedure is within the limits of variation likely to be seen in the full pragmatic trial. Treweek and Zwarenstein have argued that “if we want evidence from trials to be used in clinical practice and policy, trialists should make every effort to make their trials widely applicable, which means trials should be pragmatic in attitude” [11]. It would seem pragmatic to extend this attitude to internal pilots.

There are considerable advantages to carrying data forward from pilot trials, including: reduced cost, reduced burden on study populations, increased numbers of participants in the full trial, and maintenance of momentum from pilot to main trial [12]. However, the scientific integrity of a trial requires that the aims and methods of the pilot study, if not its geographical extent, match those of the full trial. If ‘feed forward’ of data is to take place from pilot to full trial, those charged with overseeing the adequacy of trial conduct, for example Trial Steering Committees (TSCs) and Data Monitoring and Ethics Committees (DMECs) need a structured approach to identifying and documenting any protocol developments that take place after the start of the planned internal pilot.

Within the literature on trial methodology, there are existing tools for reporting pilot trials and for defining ‘success’ in pilot studies. For example, Thabane et al. [8] highlight the importance of assessing the success of a pilot study based on predefined criteria, typically relating to recruiting and retaining participants. Their approach is valuable when assessing feasibility and acceptability of study methods, but is not focussed on decision-making relating to the appropriateness or otherwise of carrying data forward to the main trial dataset. They suggest four potential outcomes for pilot studies: stop; continue but modify protocol (feasible with modifications); continue without modifications but monitor closely (feasible with close monitoring); or continue without modification (feasible as is). Where a decision has been made to stop then there should be no carry forward of data, whereas decisions to continue without modification would allow for data carry-forward. Within the category of ‘continue but modify protocol’ (feasible with modifications) it may or may not be appropriate to carry data forward to the full trial. It is within this category that further consideration is required regarding the nature of modifications and their potential impact on the integrity of the final dataset.

In considering approaches to the ‘pooling’ of pilot data and full trial data, we have also considered ‘adaptive trials’, defined as “a design that allows modifications to be made to a trial’s design or statistical procedures during its conduct with the purpose of efficiently identifying clinical risks/benefits of a new intervention or to increase the probability of success of clinical development” [13]. In adaptive trials the design, methods and procedures of extended internal pilots of trials are assessed mid-trial with a view to implementing pre-defined change to design or statistical procedures whilst the trial is running. The approach is based on potential amendments being identified prior to the trial and cannot be used to respond to issues that are identified only during the course of the pilot. Also, difficulties arise where interim analysis are part of the review method as analysis of early data can bias the ongoing trial by focusing on whether the intervention is effective such that findings influence design rather than the reverse [14,15].

In the absence of existing recommendations for decision-making on the acceptability of carrying forward pilot data into the full trial dataset of pragmatic trials, we wanted to develop a method of recording design modifications made during the pilot phase. By creating a method for systematically documenting amendments, and for recording decisions on the significance of these amendments, we intended to provide a body of information that trialists could use to make an informed decision. Although a wide range of decisions is possible, we are envisaging three categories:

Decision A: unequivocal acceptance of pilot data: when the pilot confirms that design and methods are feasible and appropriate except perhaps for minor details. Pilot data can be carried forward to the main trial dataset.

Decision B: conditional acceptance: when the pilot confirms that design and methods are feasible and appropriate in principle but need specific refinements (decision B1); decision on whether to include pilot data in full trial dataset must be delayed until the variation in procedures for the full pragmatic trial is known, including both within and between site differences (decision B2).

Decision C: non-acceptance of pilot data: when a first pilot identifies the need for a substantial change that the pilot did not assess, even implicitly. Data from the pilot cannot be carried forward to the main trial dataset.

**Figure 1:**
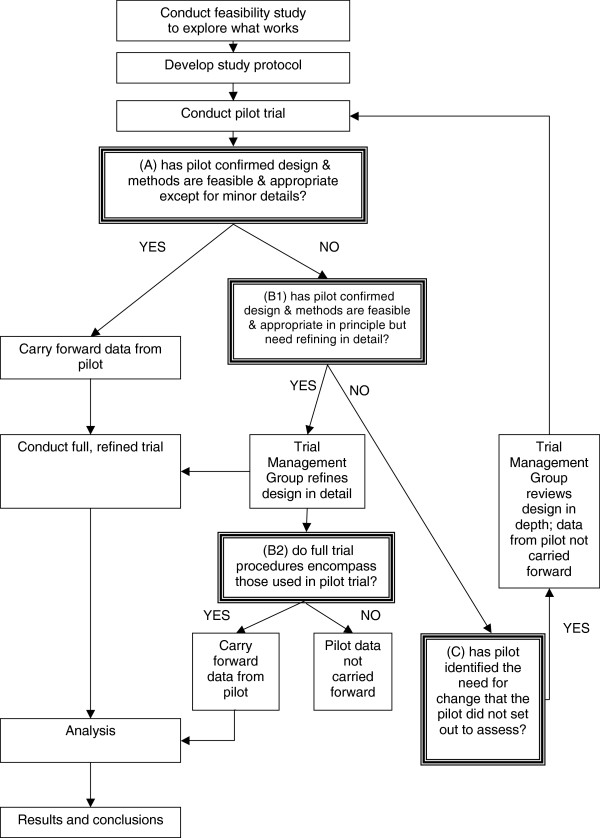
Flow chart of decision points in pilot trials.

The place of these decision points within the ‘flow’ of a trial are illustrated in Figure 1.

Two elements of the decision options above require a ‘judgement call’ by research leads or monitoring committees; for decision ‘A’ how ‘minor’ do details need to be? and; for decision ‘B’ how much procedural variation is acceptable within a full trial? The definitions of ‘minor’ versus ‘substantial’ amendments have been drawn up bodies charged with the ethical scrutiny of projects, and may prove useful in the decision making process. The degree of acceptable variation in research procedures and intervention provision within and between the sites of pragmatic trials will vary depending on the degree of heterogeneity ‘on the ground’, and the degree to which such variation can be reasonably reduced within ‘natural’ or ‘service-dictated’ provision. Methods for monitoring pilots therefore need to go beyond the issues of recruitment and retention of participants to cover all aspects of the trial.

#### Development of acceptance checklist for clinical effectiveness pilot trials (ACCEPT)

1) To ensure comprehensive coverage of pilot trial procedures we drew up a list of components derived from the CONSORT 2010 statement [16].

2) For each trial component we considered potential methods of monitoring procedures and potential outcomes from that monitoring process (see Table 1) covering:

• Feasibility and appropriateness of the trial design.

• Feasibility and appropriateness of the mechanics, management and safety of interventions.

• Acceptability and efficiency of implementing the research procedures.

**Table 1:** Acceptance checklist for clinical effectiveness pilot trials (ACCEPT): trial components, exemplar monitoring methods and exemplar outcomes

**Component of trial**		**Monitoring methods (exemplars)**	**Amend?**	**Outcomes (exemplars)**
Trial design		Review research protocol especially balance of scientific & practical needs	Yes/No	Amend trial design & dependent components. Submit amendment to Research Ethics Committee
Sample size		Test assumptions within protocol on: number of (active) centres; recruitment rates; retention rates; & SD of primary outcomes	Yes/No	Revise if necessary: sample size calculation; trial period; & funding
Interventions	Clinical governance	Assess compliance with: formal training in intervention; Health & Safety regulations; & other clinical governance requirements	Yes/No	Enhance formal training of intervention providers
	Intervention fidelity	Measure & assess adherence to intervention manual by video, observation or audio	Yes/No	Enhance clinical supervision of intervention providers
Participants	Recruitment strategy	Assess: flows of participants; cost & productivity of each route	Yes/No	Refine recruitment strategy, generally & locally
	Eligibility criteria	Assess: characteristics of sample; barriers to recruitment; update of intervention	Yes/No	Refine eligibility criteria
Consent procedures	Participant Information Sheets (PIS)	Consult participants & refusers	Yes/No	Refine PIS especially to address frequently asked questions
	Taking informed consent	Audit consent documentation. Measure & assess adherence to consent procedures by video, observation or audio	Yes/No	Enhance training of research team
Randomisation process		Check quality especially: accessibility by researchers; validity of CONSORT flowchart; & accuracy of stratifying variables	Yes/No	Refine: randomisation procedure & parameters; & training of research team
Blinding		Check whether assessors can predict individual allocations. Test whether unblinded researchers can keep other researchers blind	Yes/No	Refine blinding procedures, e.g. by reallocating responsibilities within research team
Data	Data collection	Assess adherence to interview schedules & fieldwork handbook, including duration of assessments, by video, observation or audio	Yes/No	Refine schedules to reduce assessment burden. Enhance training of research team
	Data quality	Test missing data procedures within draft analysis plan	Yes/No	Refine data collection tools & missing data procedures
	Data management	Test trial database, related procedures & link to analytical software	Yes/No	Refine trial database & procedures
Research Governance	Research protocol adherence	Enable quality assurance officer (QAO) to test adherence as widely as possible	Yes/No	Refine: protocol; quality assurance plan & training of team
	Adverse events (AE)	QAO to test procedures for: reporting AEs; assessing severity, causality & expectedness monitor at management group; report to DMEC	Yes/No	Refine AE reporting & assessment procedures
	Health & Safety	Test H&S procedures, e.g. for lone working	Yes/No	Refine H&S procedures
Data analysis		Test draft analysis plan on pilot data	Yes/No	Refine analysis plan to address research aims in full
Trial management		Review role descriptions of research team. Review remits of trial management group, trial research team etc.	Yes/No	Review role descriptions of research team. Refine roles e.g. if workloads vary. Refine remits e.g. if inadequate reporting of any component

The table of proposed trial components, monitoring methods and monitoring outcomes formed the draft ACCEPT document.

3) Based on the draft checklist we planned and tested methods of monitoring, and recorded outcomes of monitoring, for a pilot trial of psychological interventions for family carers of people with dementia [17]. We gathered evidence on design, methods and procedures from research participants, research staff, the providers of interventions (both experimental and control) and recipients of those interventions. The trial research team and trial management group, including grantholders and methodologists, DMEC and TSC, reviewed the recorded evidence. On the basis of the monitoring outcomes, decisions were made on whether pilot data could be carried forward to the main trial. (See following ‘Case Study’)

4) We wrote about the checklist, termed Acceptance Checklist for Clinical Effectiveness Pilot Trials (ACCEPT), and submitted the description for peer review.

5) On the basis of feedback from peer reviewers we reviewed our explanation of the rationale to place ACCEPT within an international context, and clarified its proposed use with the particular focus on carry forward of data from pilot to main trial.

### Case-study – Carer Supporter Programme (CSP)

The CSP trial is part of the SHIELD research programme into psychosocial interventions in dementia funded by the National Institute of Health Research. The trial compares four interventions: one-to-one peer support for family carers of people with dementia (CSP); large group reminiscence therapy for people with dementia and their family carers (Remembering Yesterday Caring Today – RYCT); CSP and RYCT in combination; and treatment as usual – neither CSP nor RYCT [17].

The pilot trial ran in two Boroughs of London, England. In the first site we focused on appropriateness and acceptability of procedures whereas the focus in the second site was on logistics and timing of the interventions in relation to the timetable for recruitment and research interviewing procedures. We devised monitoring methods in accordance with our draft checklist, and systematically recorded the outcomes of these alongside recording of the implications for the main trial.

Drawing from the trial components of eligibility criteria (a subcomponent of ‘participants’), trial design, intervention fidelity (a subcomponent of ‘interventions’) and data quality, we provide examples of the monitoring methods used, the outcome of the monitoring process and decisions made on the potential pooling of pilot and full trial data.

#### Eligibility criteria

Eligible participants were people with primary progressive dementia and their family carers. Early feedback from recruiters identified uncertainty in assessing eligibility when presentations were a-typical, where there were comorbidities such as stroke, head injury or movement disorders, and where potential participants without a formal diagnosis of dementia were nevertheless in receipt of dementia-related treatments and services. During the pilot we reviewed and clarified the screening tool, and encouraged discussion with clinical researchers where there was uncertainty. Eligibility of clients with a-typical presentations was discussed on a case-by case basis throughout the trial, but the eligibility of most potential participants could be determined on the basis of the original screening questions. We therefore concluded that changes to the assessment of eligibility criteria could be considered minor and were not a barrier to the carry forward of data from the pilot to full trial.

#### Trial design

The two psychosocial interventions had different delivery formats: CSP focused on individual carers while RYCT focused on groups of people with dementia and their carers. We therefore needed a trial design that allowed simultaneous delivery of individual interventions and a group programme such that a random sample of participants received the combined intervention. We devised a 2-stage randomisation process in which each participant was randomised individually immediately post-baseline assessment to either CSP intervention or control, and subsequently entered into a group randomisation process to either RYCT or control. Experience in the first pilot site was that the time-lag between the first and last individual randomisation was such that the proposed timetable for follow-up interviews was put in jeopardy. In the second pilot the individual intervention was yoked to the group timetable and procedures tightened such no potential participants undertook initial assessments until a minimum cohort size had been identified as willing and eligible. In the main trial there continued to be variation in the time difference between baseline assessment and the start of intervention, and between the end of intervention and final follow-up interview. The monitoring data will allow comparison between arms of the trial and a decision on whether to include pilot data in the final dataset will need to be made once final follow-ups are complete.

Also relating to trial design, feedback from researchers in the first pilot site alerted the research team to participants expressing greater interest in the individual support intervention than the group intervention. This had implications for intervention uptake and the feasibility of the group intervention sessions. Prompt discussion with the trial methodologists before the group randomisation in the first pilot site enabled us to adjust randomisation ratios for the group randomisation from 1:1 to 2:1 (RYCT:control). The revised randomisation ratios facilitated adequate RYCT group size in subsequent rounds of recruitment and intervention. If this modification had not been made prior to the first group randomisation, data from the first pilot site could not have been carried forward to the main trial dataset as the trial design of the pilot would have differed significantly from that used in the main trial.

#### Intervention fidelity

In order to ensure intervention fidelity, experienced clinicians in the research team were closely involved in the set up and provision of interventions at the pilot stage. Recording systems on aspects of intervention fidelity were devised and tested during the pilot and then implemented fully during the full trial. Variation of intervention provision was greater during the full trial than it had been in the pilot sites due to differences in the funding and organisation of services in each geographical area. For example, in the trial sites the RYCT intervention was delivered with involvement from National Health Service (NHS) employees and the Carer Supporter intervention provided by organisations within the voluntary and charitable sector. This model of intervention provision was replicated for most rounds of recruitment and intervention for the full trial but in some additional sites both interventions were provided by the NHS and in one further site a voluntary/charitable sector organisation provided both. The same RYCT group trainer was used throughout the trial, and, although minor details of the content of training were amended through the course of the trial, the core of the training manual was consistent throughout the pilot and full trial. Intervention fidelity was higher in the pilot sites, but fell within the ‘envelope’ of variation seen across the full trial. A decision was therefore made that pilot data should not be held back from being pooled with the main trial dataset on grounds of variation in intervention fidelity.

#### Data quality

An early audit of the completeness of baseline data in the first pilot site enabled us to identify systematically missing data. The main issue was the formatting of data collection tools, which allowed researchers or respondents to misinterpret question routing and omit questions. Hence we reformatted these tools and re-trained researchers. The corresponding audit in the second pilot site showed a reduction in missing data. Thus early identification of data collection issues, and immediate establishment of monitoring, enhanced the quality of the dataset throughout the trial, and kept the proportion of missing data to a minimum. A high proportion of missing data would have led to a decision to exclude pilot data from the main trial dataset.

## Conclusions

Using ACCEPT enabled us to systematically record trial monitoring methods, and record challenges and changes to the design, methods and procedures of the trial, together with working solutions for a pragmatic trial of two complex psychosocial interventions. Transparent recording of monitoring methods and outcomes facilitated decision-making on whether data could be appropriately pooled with the main trial dataset.

Consistent with guidelines for reporting pilot trials [8], we have based ACCEPT on the trial elements highlighted in the CONSORT 2010 statement [16]. The systematic recording and reporting of monitoring methods and of changes in, and deviations from, the original protocol is a requirement of ethical committees and CONSORT reporting standards [16]. Decisions on whether to ‘carry forward’ data from the pilot trial to the main trial will depend on the degree of acceptable variation in procedures within the main trial. This will be influenced by the location of the trial along the pragmatic-explanatory continuum [18]. The final decision on inclusion of pilot data may not be made until after final data collection as although some components (e.g. trial design) need only a brief, defined pilot period, other components (e.g. data analysis) benefit from an extended pilot period.

In line with guidance for developing health research reporting guidelines [19], ACCEPT needs testing with other designs in other contexts. Fortunately the focus on design, methods and procedures facilitates the tailoring of ACCEPT to individual trials. Furthermore it permits tracking of progress by the trial management group, DMEC and TSC. Above all ACCEPT provides a systematic basis for decisions about whether or not a pilot has adequately tested a trial’s design, methods and procedures, and thus whether pilot data can be integrated into the main trial.

## Abbreviations

ACCEPT: Acceptance checklist for clinical effectiveness pilot trials; AE: Adverse events; CONSORT: Consolidated standards of reporting trials; CSP: Carer Supporter programme; DMEC: Data monitoring and ethics committee; H&S: Health and safety; NHS: National health service; NIHR: National institute of health research; PIS: Patient information sheet; QAO: Quality assurance officer; RYCT: Remembering yesterday, caring today; SHIELD: Support at home: interventions to enhance life in dementia; TSC: Trial steering committee.

## Competing interests

The authors declare that they have no competing interests.

## Authors’ contributions

IR conceived the notion of a pilot acceptance plan. GC and KB developed such a plan for the CSP pilot trial and implemented it successfully. GC, KB, MO and IR drafted the paper. All authors commented on successive drafts. GC produced the final version. All authors read and approved the final manuscript.

## Pre-publication history

The pre-publication history for this paper can be accessed here:

http://www.biomedcentral.com/1471-2288/13/78/prepub
